# Complete Genome Sequences of Curli-Negative and Curli-Positive Isolates of Foodborne *Escherichia coli* O157:H7 Strain 86-24

**DOI:** 10.1128/genomeA.01323-16

**Published:** 2016-12-15

**Authors:** Vijay K. Sharma, Darrell O. Bayles, David P. Alt, Torey Looft

**Affiliations:** a Food Safety and Enteric Pathogens Research Unit, National Animal Disease Center, ARS-USDA, Ames, Iowa, USA; b Infectious Bacterial Diseases Research Unit, National Animal Disease Center, ARS-USDA, Ames, Iowa, USA

## Abstract

*Escherichia coli* O157:H7 strain 86-24 does not produce curli fimbriae, but gives rise to curli-positive isolates at a variable frequency. Here, we report the complete genome sequences of curli-negative and curli-positive isolates of strain 86-24.

## GENOME ANNOUNCEMENT

*Escherichia coli* O157:H7 (O157) is an organism that causes foodborne diarrheal illnesses of varied spectra. The O157 strain EDL933 (1) genome has been used as a reference for studying other O157 strains. The genomes of O157 isolates (1, 2) are larger than the *E. coli* K12 strain MG1655 genome, a fact attributed to lateral gene transfer. This genome expansion correlates with broader host range and more robust environmental survival of O157. Population heterogeneity in O157 confers genotypic variants with advantages in colonization and survival in diverse niches (3–5). Here, we describe the complete genome sequences of a curli-negative (NADC 6564) and a curli-positive (NADC 6565) isolate of strain 86-24 linked to the 1986 Walla Walla (WA) outbreak (6, 7), and identify genetic differences responsible for the curli-positive phenotype.

Genomic DNA, prepared from midlogarithmic-phase bacterial cultures (Qiagen Genomic-tip G100), was used for generating 20-kb large-insert libraries and sequenced using the PacBio RS II platform (Yale Center for Genomic Analysis, West Haven, CT). The PacBio reads were assembled into contigs (PacBio SMRT Analysis 2.3.0 and CANU v. 1.3) (8). For Illumina MiSeq sequencing, genomic DNA libraries were prepared using the TruSeq Nano DNA Library Preparation kit. The PacBio assemblies were circularized using the AMOS v. 3.1.0 Minimus2 (9) assembler and the genomes subjected to one round of error correction and polishing with the Quiver algorithm by remapping the PacBio reads to their respective circularized genomes as the references. These assemblies were further error corrected and polished to their final version by mapping the Illumina reads to their respective genomes and performing consensus recalling and corrections using the Broad Institute’s Pilon v 1.18 (10). Both genomes were broken to begin at the *dnaA* gene. The fully closed genomes and associated plasmids were annotated by the NCBI Prokaryotic Genome Annotation Pipeline.

The NADC 6564 genome consisted of a chromosome of 5,466,770 bp and a plasmid of 92,691 bp, contained a total of 5,676 predicted genes (5,578 for the chromosome and 98 for the plasmid), 5,542 coding sequences (CDS) including pseudogenes and 5,399 genes. The NADC 6565 genome comprised a chromosome of 5,467,107 bp and a plasmid of 92,690 bp, contained a total of 5,678 predicted genes (5,580 for the chromosome and 98 for the plasmid), 5,544 CDS including pseudogenes and 5,404 genes. Both genomes contained 103 tRNA, eight 5S RNA, seven each of 16S and 23S RNA, and nine noncoding RNA (ncRNA) genes and had G+C content of 50% and 48% for the chromosomes and plasmids, respectively. There were 143 predicted pseudogenes in NADC 6564 and 140 in NADC 6565. The NADC 6564 and NADC 6565 genomes aligned (Artemis Comparison Tool) (11) with the EDL933 genome except for some yet-uncharacterized inversions and indels. A tandem duplication of a 5-bp sequence was identified in *rcsB* of the *rcsBD* signal transduction system (12) and was determined to confer the curli-positive phenotype.

### Accession number(s).

The genome sequences have been deposited at GenBank under the GenBank accession numbers CP017251 and CP017252 (NADC 6564) and CP017249 and CP017250 (NADC 6565).
